# Exploring the Use of Cytochrome Oxidase c Subunit 1 (COI) for DNA Barcoding of Free-Living Marine Nematodes

**DOI:** 10.1371/journal.pone.0013716

**Published:** 2010-10-28

**Authors:** Sofie Derycke, Jan Vanaverbeke, Annelien Rigaux, Thierry Backeljau, Tom Moens

**Affiliations:** 1 Marine Biology Research Group, Department of Biology, Ghent University, Ghent, Belgium; 2 Centrum for Molecular Phylogeny and Evolution, Ghent University, Ghent, Belgium; 3 Joint Experimental Molecular Unit, Royal Belgian Institute of Natural Sciences, Brussels, Belgium; 4 Evolutionary Biology Group, Department of Biology, University of Antwerp, Antwerp, Belgium; California Academy of Sciences, United States of America

## Abstract

**Background:**

The identification of free-living marine nematodes is difficult because of the paucity of easily scorable diagnostic morphological characters. Consequently, molecular identification tools could solve this problem. Unfortunately, hitherto most of these tools relied on 18S rDNA and 28S rDNA sequences, which often lack sufficient resolution at the species level. In contrast, only a few mitochondrial COI data are available for free-living marine nematodes. Therefore, we investigate the amplification and sequencing success of two partitions of the COI gene, the M1-M6 barcoding region and the I3-M11 partition.

**Methodology:**

Both partitions were analysed in 41 nematode species from a wide phylogenetic range. The taxon specific primers for the I3-M11 partition outperformed the universal M1-M6 primers in terms of amplification success (87.8% vs. 65.8%, respectively) and produced a higher number of bidirectional COI sequences (65.8% vs 39.0%, respectively). A threshold value of 5% K2P genetic divergence marked a clear DNA barcoding gap separating intra- and interspecific distances: 99.3% of all interspecific comparisons were >0.05, while 99.5% of all intraspecific comparisons were <0.05 K2P distance.

**Conclusion:**

The I3-M11 partition reliably identifies a wide range of marine nematodes, and our data show the need for a strict scrutiny of the obtained sequences, since contamination, nuclear pseudogenes and endosymbionts may confuse nematode species identification by COI sequences.

## Introduction

Free-living nematodes dominate marine sediments both in terms of densities (10^5^–10^7^ individuals m^−2^) and diversity (>10 species cm^−2^) [1]. They play an important role in benthic food webs where they are a high quality food source for higher trophic groups [2] and at the same time influence the composition of lower trophic groups [3], [4]. Nevertheless, the study of free-living marine nematodes is held back because their morphological identification is notoriously difficult. This is due to the paucity of diagnostic characters and the fact that these characters are often doubtful to score and interpret, particularly when relying on traditional light microscopy [5]. Therefore, nematode communities are usually only surveyed up to genus rather than species level. This may be problematic, because functional roles of nematodes may be highly species-specific [3], [6] and their population dynamics can be affected by the presence of closely related species, often congeners [7]–[9]. Hence, the identification of nematodes could greatly benefit from the use of molecular tools, as these may provide a faster and more reliable estimate of nematode diversity [10]–[12]. Such molecular studies typically use the 18S rDNA, mainly because of the availability of universal nematode primers and its phylogenetic resolution at the genus and higher taxon level [5]. Unfortunately, the 18S gene has low resolution when it comes to distinguishing closely related species [5], [13]–[15].

The mitochondrial cytochrome oxidase c subunit 1 (COI) gene is one of the most popular markers for population genetic and phylogeographic studies across the animal kingdom [16]. Its popularity has increased even more since it appears that the M1-M6 partition of the COI gene (hereafter referred to as the Folmer region) is an efficient identification tool for Metazoan species, turning it into the core fragment for DNA barcoding [17]. Nevertheless, COI based DNA barcoding sometimes faces problems: (1) in some taxa, such as Porifera, Anthozoa, fungi, plants [18]–[20], the Folmer region shows little resolution at the species level so that other COI regions such as I3-M11 [21], or other genes such as the nuclear ribosomal ITS [22] have been proposed for barcoding purposes, and (2) the occurrence of nuclear copies of the COI gene (so-called ‘numts’) may confuse DNA barcoding results and may lead to an overestimation of taxonomic diversity [23]. For marine nematodes, COI based DNA barcoding is problematic because the ‘universal’ invertebrate M1-M6 primers [24] generally give very poor amplification results [5], [10], while the I3-M11 primers amplify satisfactorily in three nematode species complexes belonging to different families, viz. Monhysteridae [25], Rhabditidae [26], Leptosomatidae [15]. Furthermore, the I3-M11 partition proved its taxonomic utility by uncovering cryptic diversity in both parasitic [27], [28] and marine free-living nematodes [25], [29]. It remains, however, unclear to what extent the I3-M11 partition can be amplified more generally in other marine free-living nematodes and thus can be used as a more general DNA barcoding fragment in this group.

Against this background, we here compare the amplification and sequencing success of a modified version of the COI primerset for the I3-M11 fragment developed by Bowles et al. [27] with that of the universal invertebrate Folmer primers for the M1-M6 fragment, in 41 marine nematode species representing all marine orders dealt with in Meldal et al. [14]. To this end, nematodes were morphologically identified to species level, their morphology was video vouchered and sequences of both COI partitions were (1) checked against Genbank and used for constructing a neighbor joining tree to verify their nematode origin, (2) subjected to a strict quality control, and (3) used to construct frequency distributions of intra- and interspecific genetic distances to detect barcoding gaps.

## Materials and Methods

### Specimen collection

Nematodes were collected during upcoming tide at four intertidal locations along the Westerschelde estuary (The Netherlands) and in one coastal location (Nieuwpoort, Belgium) on April 22^nd^ 2009 (Table 1). Three perspex cores (10 cm^2^) were randomly placed in the sediment midway between the low and high water line. The uppermost two centimeters of sediment were pooled for each location. In the lab, nematodes were removed from the sediment by decantation, and the nematodes were rinsed off a 38 µm sieve with artificial sea water (Instant Ocean® salt, Aquarium Systems, France). Subsequently, living specimens were screened under a dissecting microscope, and nematodes with different morphological features and/or different behavior were handpicked, temporarily mounted in a microcompressor slide (Taylor's Microcompressor Mk II; Taylor, 1991) and heat killed. Identification to species or genus level was done by an expert nematode taxonomist (J.V.) using a LEICA DMR research microscope. In addition to the freshly collected field specimens, six marine nematode species from permanent lab cultures were added. Digital photographic vouchers representing head and tail regions of each specimen were taken at small, intermediate and immersion oil magnification. Immediately after the vouchering procedure, nematodes were collected from the temporary slide, put in lysis buffer and stored at −20°C until further processing.

**Table 1:** Primer sequences for amplification of the I3-M11 partition in marine nematodes.

Primer	Sequence (5′-3′)	Position	Source
JB3 (F)	TTT TTT GGG CAT CCT GAG GTT TAT	2179	Bowles *et al.* 1992
JB4.5 (R)	TAA AGA AAG AAC ATA ATG AAA ATG	2597	Bowles *et al.* 1992
JB5 (R)	AGC ACC TAA ACT TAA AAC ATA ATG AAA ATG	2597	Derycke *et al.* 2005
JB2 (F)	ATG TTT TGA TTT TAC CWG CWT TYG GTG T	2201	Derycke *et al.* 2007
JB2s3 (F)	ATG TTT TGA TTT TAC CWG SWT TTG G	2201	this study
JB5GED (R)	AGC ACC TAA ACT TAA AAC ATA RTG RAA RTG	2597	Derycke *et al.* 2007
JB7GED (R)	ATC AGG ATA ATC CAA ATA YTT WCG WGG	2780	this study

(Primer): name of the primer. (F) forward primer; (R) reverse primer. (Sequence): primer sequence. (Position): starting position of each primer along the COI sequence of *Drosophila yakuba*. (Source): publication of the primer.

### I3-M11 primer development

Two I3-M11 primer sets were constructed based on an existing primer set developed for the parasitic platyhelminth *Echinococcus granulosus* ([27], Table 1). Initially, the unmodified primer set worked well for *Rhabditis* (*Pellioditis*) *marina*, except for one population [29]. We therefore modified the reverse primer based on an alignment of complete COI sequences from mostly parasitic nematodes available from Genbank (Accession numbers AF538716.1, AY265417.1, AJ537512.1, AJ556134.1, AJ417719.2, AF015193.1, AY591323.1, AJ558163.1, X54252.1). This modified primer set (JB3 and JB5, Table 1) has successfully been used in rhabditid and leptosomatid nematode species [15], [26], but was unable to amplify the I3-M11 fragment in the monhysterid *Halomonhystera disjuncta* [25]. For this species complex, we then developed a degenerated primer set (JB2-JB5 GED, Table 1) based on an alignment with the Genbank sequences we had downloaded before, the rhabditid sequences we had by then, and one *Halomonhystera* sequence we had obtained by using a reverse primer further downstream (JB7GED, Table 1).

### DNA extraction and amplification of the I3-M11 and Folmer partitions

Proteinase K (1 µl of 10 mg/ml) was added to the tubes containing a single nematode in 20 µl lysis buffer (50 mM KCl, 10 mM Tris pH 8.3, 2.5 mM MgCl_2_, 0.45% NP40, 0.45% Tween 20), followed by incubation at 65°C for one hour and at 95°C for 10 min. From each species, one specimen was randomly chosen to test the amplification success of the JB3-JB5 and JB2-JB5GED primer sets. PCR cycling conditions were: initial denaturation of 5 min at 94°C, 5 cycles of (94°C for 30 s; 54°C for 30 s and temperature decreasing with 1°C for each cycle; 72°C for 30 s) followed by 35 cycles of (94°C for 30 s; 50°C for 30 s; 72°C for 30 s), and a final extension of 10 min at 72°C. Reactions were performed for each primer set separately in total volumes of 25 µl containing 2.5 µl of 10x PCR buffer with 15 mM MgCl_2_, 2 µl of MgCl_2_ 25 mM, 0.5 µl dNTP (10 mM), 0.125 µl of each primer (25 nM), 0.125 µl TopTaq DNA polymerase (Qiagen), 18.625 µl sterile distilled water and 1 µl DNA. For the degenerated primer set JB2-JB5GED, 0.5 µl of each primer (25 nM) was used. In our experience, the TopTaq DNA polymerase (Qiagen) outperforms Taq DNA polymerase (Qiagen) and DyNAzyme EXT DNA polymerase (New England Biolabs) as it yields larger amounts of PCR product.

Amplification of the Folmer region was done using primer sets LCO1490 – HCO2198 [24] following the PCR protocol published on http://barcoding.si.edu/.

PCR products were loaded on 1% agarose gels containing 0.003% EtBr and visualized using BioDoc-It ™ Imaging System (UVP). Each gel contained one lane with 5 µl of Low DNA Mass Ladder (Invitrogen), while all other lanes contained 5 µl PCR product which had been mixed with 1 µl of loading dye. Amplifications were considered successful when a band of the expected size was observed on agarose gel. Samples showing the correct band together with aspecific products were also considered successful.

### Sequencing

PCR products were enzymatically cleaned with calf intestine alkaline phosphatase (1 U µl^−1^, Fermentas) and exonuclease I (20 U µl^−1^, Fermentas) for 15 min at 37°C followed by 15 min at 85°C. Cycle sequencing was performed with the ABI Prism BigDye V 3.1 Cycle Sequencing kit (Applied Biosystems) on an ABI Prism 3130XL capillary sequencer, in both directions using the same primers as for the PCR. Chromatograms were assembled in DNASTAR Lasergene SeqMan Pro v.7.1.0. Forward sequences for the I3-M11 partition sometimes showed double peaks or a low signal. A new forward primer was developed which was similar to JB2: JB2s3 is three bp shorter at the 3′ end and has other degenerated positions than JB2 (Table 1). Unidirectional sequences were considered successful when high quality chromatograms (i.e. no double peak patterns and high fluorescence signal) were obtained for at least 200 bp. Sequencing success for each primer was calculated by dividing the number of successful sequencing reactions by the total number of sequencing reactions performed for that particular primer. Sequencing success for each partition was calculated by dividing the sum of successful sequencing reactions of the forward and reverse primer by the sum of the total number of sequencing reactions performed for each primer.

### Sequence quality control

The nematode origin of sequences was first investigated by a blastx search against the non- redundant nucleotide database in Genbank. In view of the low number of nematode COI sequences available and the high sequence divergence between distantly related nematode species, identity matches with nematodes or with any other organism were generally lower than 80%. Consequently, COI sequences were validated by constructing a phylogenetic tree: sequences from nematodes were expected to reflect known phylogenetic relationships among closely related marine nematode species. All sequences were translated using the invertebrate mtDNA genetic code in Seaview v 4.1 [30] and aligned using Muscle [31] as implemented in Seaview v 4.1. Although the mtDNA genetic code of nematodes may differ from that of the invertebrate translation code [32], this is unlikely to affect our inferences since Jacob *et al.* [32] showed that a stopcodon was changed into a tyrosine codon, and not the other way around. The nucleotide alignment was subsequently used to construct a neighbor joining (NJ) tree in MEGA v 4.0 [33] using the K2P-model. Although this is not the optimal substitution model for our data, it is the generally used model for DNA barcoding and for inferring barcoding gaps [34]. Finally, a quality check of the sequences was performed as suggested by Song et al. [23]: sequence chromatograms were investigated for the presence of double peaks without indication of additional products on agarose gel, translated sequences were checked for the presence of frame-shift mutations or stop codons and nucleotide and amino acid composition was calculated in Mega v 4.0.

### Intra- and interspecific genetic distances

Pairwise sequence divergence using the K2P-substitution model was calculated in MEGA v 4.0. Intraspecific and congeneric K2P distances were calculated using all I3-M11 sequences from three previous population genetic studies [15], [25], [26], while K2P distances between species from different genera were calculated using the species listed in Table S1.

## Results

### Specimens collected

In total, 102 specimens were screened, yielding 41 species belonging to 33 genera (Table S1, 1–41), representing all families, subordos or ordos involving marine taxa as indicated by Meldal *et al.* [14], except for the subordo Desmoscolecida, of which we had no specimens. For each of the 41 species, one specimen was used to assess the amplification and sequencing success of I3-M11 and M1-M6. Specimens yielding a PCR product of the expected size and without aspecific products smaller than the expected PCR product were sequenced. For the I3-M11 partition, we added six genera (*Rhabditis*, *Halomonhystera*, *Thoracostoma*, *Pseudocella*, *Deontostoma and Phanoderma*) from previous studies (Table S1, numbers 42–65), resulting in a total of 38 genera.

### Amplification and sequencing success of the I3-M11 partition

**Figure 1:**
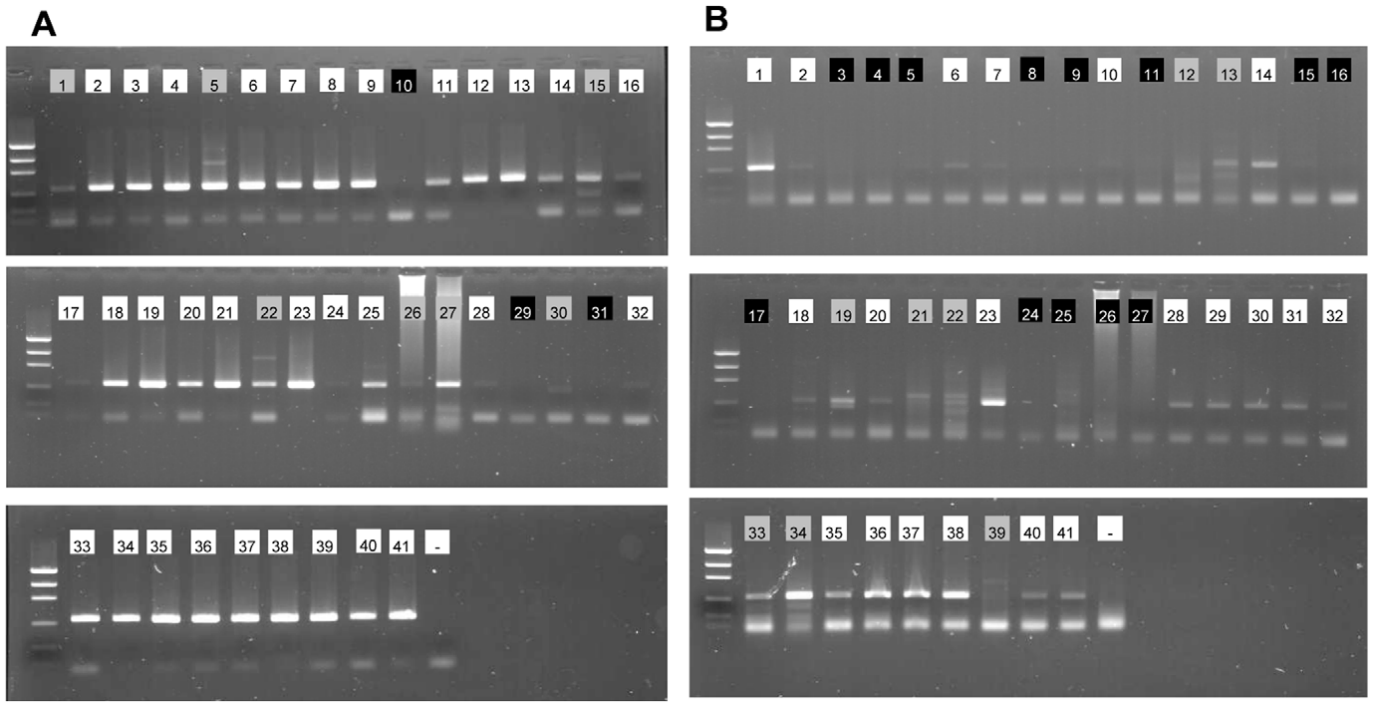
I3-M11 partition. PCR products of 41 marine nematode species using 0.125 µM of primers JB3-JB5 (A) or 0.5 µM of primers JB2-JB5GED (B). Numbers in lanes correspond to the numbers in Table S1, background colours of the numbers indicate quality of PCR product: white  =  band of expected size and no aspecific products, grey  =  aspecific bands, black  =  no product. -  =  negative control.

The JB3-JB5 primer set clearly outperformed the degenerated JB2-JB5GED primer set in terms of amplification success and lack of aspecific products (Fig 1). Of the 41 species tested, three (7.3%) vs. 13 (31.7%) did not produce any products, respectively. Aspecific products were formed in seven (17.0%) vs. eight species (19.5%), respectively (Fig 1). Hence, amplification success was 87.8% vs. 53.6%, respectively. Interestingly, both primer sets were more or less complementary: species with weak or no amplification for primer set JB3-JB5 (samples 1, 28, 29, 30, 31, 32) generally produced stronger bands when the degenerated primer set was used (Fig 1). In view of the high amplification success of JB3-JB5, we used this primer set in those species for which we had more than one specimen (see Table S1). Many species showed a consistent amplification with strong bands, except *Ascolaimus*, *Eleutherolaimus* and *Bolbolaimus* for which amplification was relatively weak.

The I3-M11 partition was sequenced with JB3 and JB5 for 34 species. Despite the very high amplification success of the I3-M11 partition, sequencing success was 63.4% using the JB3-JB5 primer set. Reverse sequences generally produced more unambiguous chromatograms than forward sequences (73% and 56%, respectively). The ambiguous forward sequences showed double peaks in the first 200 bp (two samples), after 300 bp (five samples) or over the whole length of the sequence (three samples). The lower number of ambiguous JB5 sequences in combination with shorter double peak fragments of approximately 200 bp in the JB3 sequences suggests that for some nematode species the JB3 primer may also bind further downstream in the I3M11 partition. No improvement of the forward sequences was obtained when the annealing temperature of the sequencing reaction was increased to 54°C, or when the JB2 primer was used to sequence the PCR product. Finally, we developed a new primer JB2s3 to sequence, and this improved the chromatograms in four out of six problematic samples tested. In total, we obtained 31 bidirectional sequences with JB3-JB5: sequencing failed for *Southernia* and *Adoncholaimus* (indicated by ‘-’in Table S1) and for *Crenopharynx* the forward sequence failed (indicated by R in Table S1). For JB2-JB5GED, we sequenced only *Onyx* and *Bolbolaimus* (indicated in bold in Table S1), since we already sequenced *Halomonhystera*, *Diplolaimella* and *Diplolaimelloides* in a previous study [35]. In this way, we obtained 33 new sequences for the I3M11 partition.

### Amplification and sequencing success of the Folmer partition

**Figure 2:**
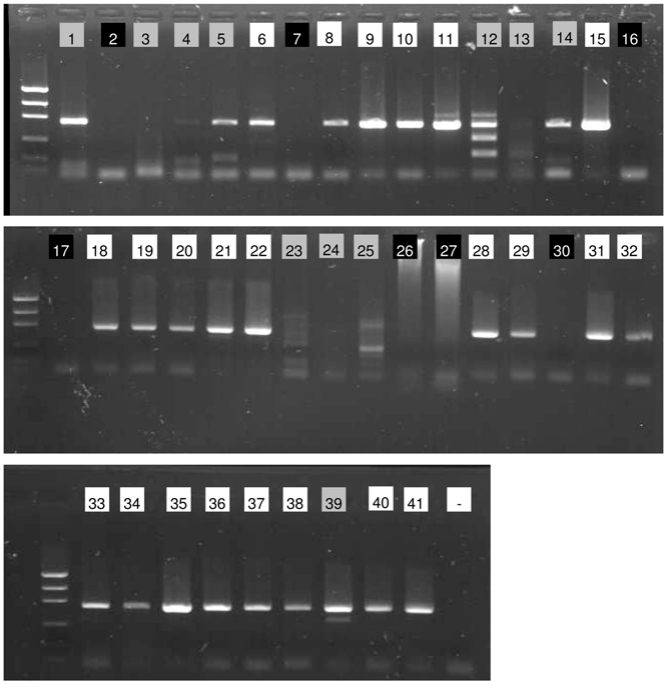
Folmer partition. PCR products of 41 marine nematode species using 0.125 µM of primers LCO1490 and HCO2198. Numbers in lanes correspond to the numbers in Table S1. Background colours of the numbers indicate quality of PCR product: white  =  expected band, grey  =  aspecific bands, black  =  no product. -  =  negative control.

The amplification success with the Folmer primers was 65.8%. No amplification was observed in seven species and aspecific products were formed in 11 species (Fig 2).

The Folmer partition was sequenced in both directions for 28 species (Table S1) with a success rate of 63.8%. Forward and reverse primers had approximately equal sequencing success (62.0% and 65.5%, respectively). For *Dichromadora microdonta*, only the reverse sequence gave a good signal. In total, we obtained 18 bidirectional sequences with LCO1490-HCO2198.

### Sequence quality control

**Figure 3:**
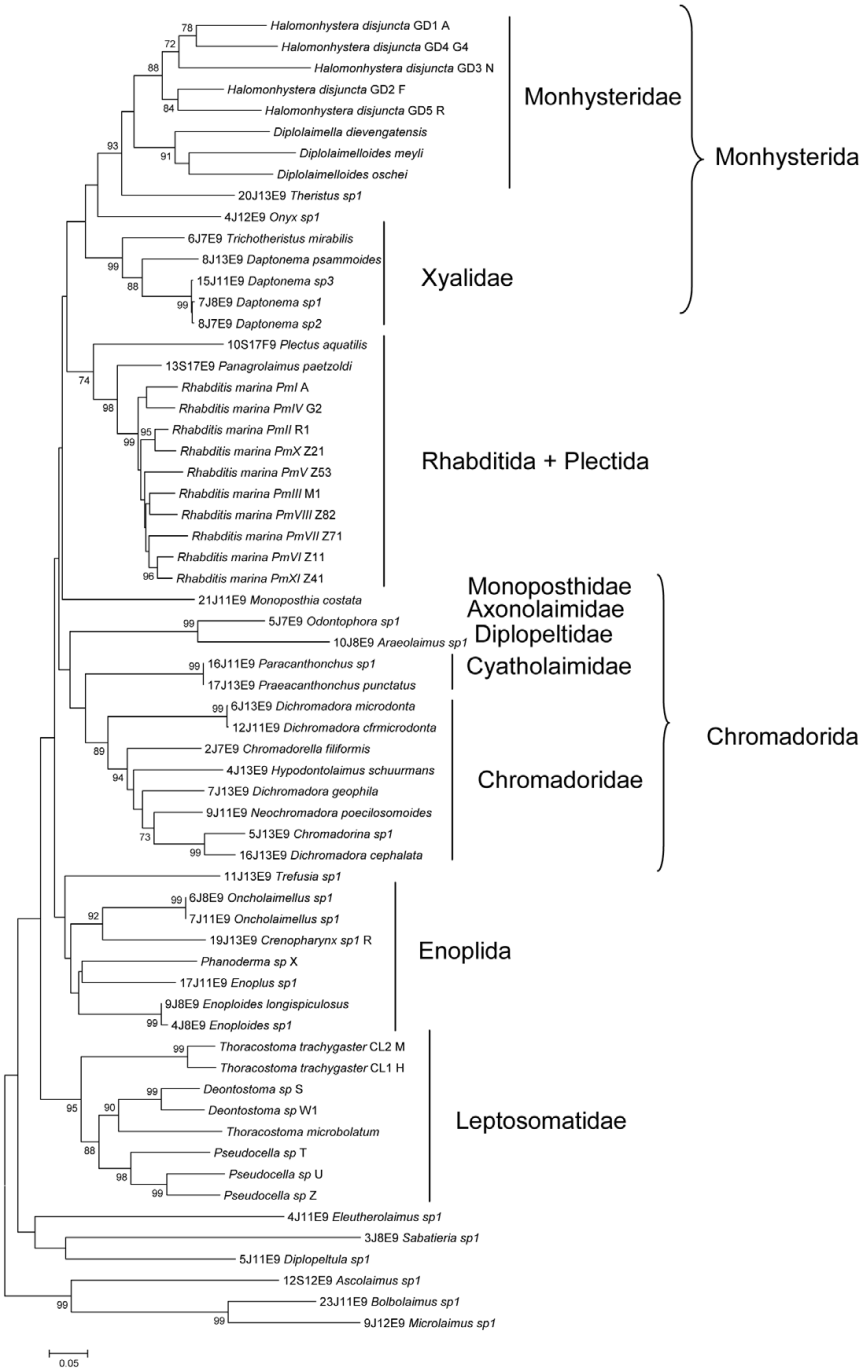
NJ-tree of the I3-M11 partition based on K2P genetic distance. Sequences with voucher number are from this study, sequences without voucher numbers are from previous population genetic studies. Higher taxon levels are indicated after the vertical lines and brackets.

Assembled sequences were subsequently compared with the Genbank database to check their nematode origin. All hits reported hereafter had a coverage of 99% or 100%. For the I3-M11 partition, two cases showed a similarity higher than 85% with gamma-proteobacteria: *Bolbolaimus* (86% similarity) and *Microlaimus* (94% similarity). Three sequences did not show a single hit with nematodes, and instead showed low similarity with sea urchin (58% *Eleutherolaimus*), flagellates (70% *Ascolaimus*), and beetles (76% *Monoposthia*). All other sequences were most similar to nematodes, but with most values being less than 80% (similarity range between 61% and 94%). No stop codons or frame shift mutations were observed in the alignment. Six sequences contained indels: *Ascolaimus*, *Bolbolaimus* and *Microlaimus* sequences showed a deletion of one amino acid at position 19, the *Eleutherolaimus* sequence showed two insertions at positions 87 and 88 and *Praeacanthonchus/Paracanthonchus* sequences showed a large insertion of nine amino acids compared to all other sequences in the alignment. The latter two species had identical I3-M11 sequences. To exclude methodological errors, we sequenced all specimens of both species (5 and 4, respectively, Table S1), and all nine sequences were identical. The NJ tree generally showed a topology congruent with that of known families and ordines of marine nematodes (Fig 3), except for six species which had particularly long branches and positioned closer to the basal node in the NJ tree: *Ascolaimus*, *Bolbolaimus*, *Microlaimus*, *Diplopeltula*, *Sabatieria* and *Eleutherolaimus* were expected to cluster within the Chromadorida clades. In view of their basal position, the particularly long branches, the high similarity with bacteria or metazoan organisms and the weak amplification in other specimens, we removed all of them from the dataset for all subsequent analyses. Consequently, from the 41 species that were tested, we obtained 27 high quality sequences with the JB3-JB5 primerset (65.8%). This value increases up to 76.6% when including the 19 species from previous studies (Table S1).

**Figure 4:**
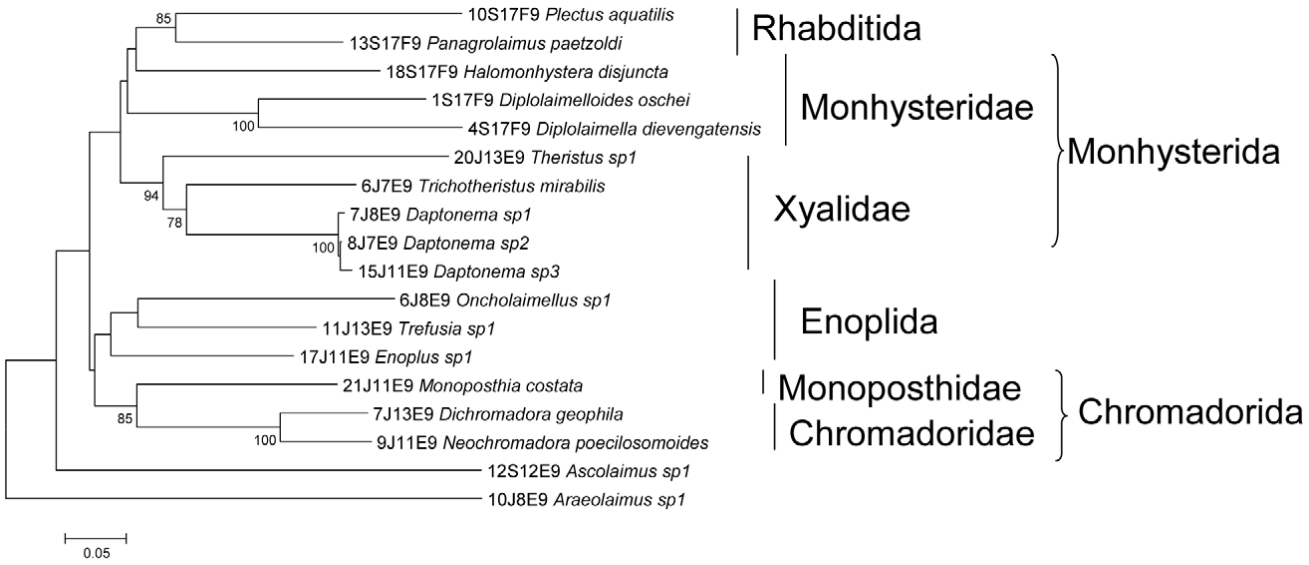
NJ-tree of the Folmer partition based on K2P genetic distance. Higher taxon levels are indicated after the vertical lines and brackets.

For the Folmer partition, Blastx searches indicated that only the *Panagrolaimus* and *Plectus* sequences showed similarity to nematodes (91% and 72%, respectively). All other sequences showed blast hits with low similarities (65% to 76%) to a variety of organisms such as polychaetes, flatworms, spiders and wasps. The alignment showed one amino acid deletion in *Panagrolaimus* (position 119) and in *Theristus* (position 159) and three deletions in *Araeolaimus* (positions 162, 163 and 206). The NJ tree generally was congruent with known taxonomy, except for *Ascolaimus* and *Araeolaimus*, which showed long branches positioned closer to the basal node (Fig 4). These sequences were removed from the dataset for calculation of genetic distances. Consequently, from the 41 species that were tested, we obtained 16 high quality sequences (39.0%).

### Variability of the I3-M11 and Folmer partition

**Figure 5:**
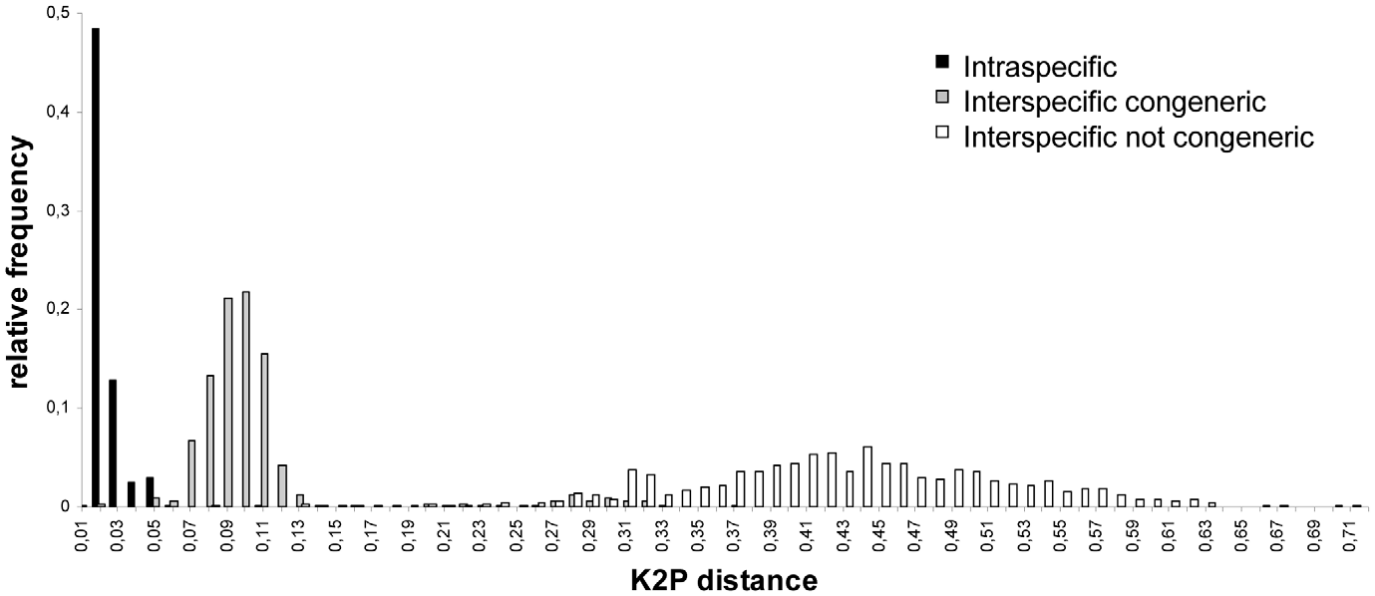
Relative frequencies of K2P-genetic distances within species (black), between congeneric species (grey) and between species from different genera (white).

Variability of I3-M11 was calculated using 27 sequences from the present study and 24 sequences from previous studies (Table S1). The I3-M11 partition was highly AT-rich (A: 27.1%, T: 42.8%, G: 18.6%, C: 11.5%). A very high variability was observed at the amino acid level with 95 out of 143 amino acids (66.4%) being variable (Table 2). Maximum pairwise K2P-distances within species varied between 0.005–0.121, while minimum K2P-distances between congeneric species ranged between 0.005–0.26 and minimum K2P-distances between species from different genera was 0.12. Although this suggests a strong overlap between intra- and interspecific genetic distances, the frequency distribution of the K2P-distances showed that 99.5% of all intraspecific comparisons showed genetic distances of less than 5%, while 99.3% of all interspecific comparisons were higher than 5% (Fig 5). The only species pair that was less than 5% different was *Praeacanthonchus* and *Paracanthonchus*.

**Table 2:** Variability of the Folmer and I3M11 partitions.

	Folmer	I3-M11	
	16 sequences	16 sequences	54 sequences
Sequence length	468–657	367–393	288–420
Alignment length	657	393	429
nucleotide variable sites ratio	0.68	0.638	0.68
amino acid variable sites ratio	0.64	0.565	0.66
K2P distance codon position 1	0.005–0.599	0.000–0.499	0.000–0.726
K2P distance codon position 2	0.000–0.297	0.000–0.300	0.000–0.427
K2P distance codon position 3	0.019–1.458	0.016–1.469	0.000–2.430

For the I3M11 partition, the variability was calculated using the same 16 specimens for which we obtained high quality sequences for the Folmer partition, and using the complete dataset with all 54 high quality sequences.

The Folmer dataset contained sequences from 16 different marine species and had a similar nucleotide composition (A: 26.0%, T: 41.8%, G: 18.4%, C: 13.8%) and amino acid variability (141 out of 219 amino acids, 64.3%) as the I3-M11 dataset. In general, the variability of both partitions was very similar (Table 2). In view of the small dataset, we were unable to calculate intraspecific or interspecific congeneric K2P-distances. Interspecific K2P-distances between species from different genera ranged from 0.01–0.59.

## Discussion

The molecular identification of marine nematodes is rapidly advancing and already uses second generation sequencing techniques to overcome the taxonomic hurdles and time consuming extraction methods of meiofaunal organisms [12], [36]. These metagenetic studies are using ribosomal nuclear genes such as 18S and 28S because of the difficult amplification of the mitochondrial COI gene [10]–[12], [36]. The present study shows that the two most popular partitions of the COI gene have quite different amplification success in marine nematodes. Although the Folmer primers have successfully been used in a wide range of animals [34], including parasitic nematodes [37], their low amplification success for free-living nematodes is a well known problem [5], [10] and is mainly caused by the high nucleotide variability and indels at the primer sites [36]. Although the mitochondrial COI gene is highly AT rich and shows high levels of nucleotide variation even at second codon positions (Table 2), our data show that primers can be developed, such as JB3 and JB5, which clearly perform better than the universal Folmer primers. Furthermore, the COI gene is able to differentiate almost all nematode species tested in the present study. Similar results were obtained in a barcoding study on parasitic nematode species [38] where taxon specific primers were used to amplify ca 550 bp of the Folmer and I3-M11 partitions [39]. These primers did not amplify well in our marine specimens, illustrating that different primer sets will be required when one wants to capture the whole diversity of nematodes.

The COI gene discriminated all morphological species, except for *Paracanthonchus/Praeacanthonchus*: although the distinction of the two genera is subtle and species from the latter genus formerly were placed in *Paracanthonchus*, the specimens identified here are morphologically distinct and should therefore show distinct I3-M11 sequences. The identical COI sequence may be the result of mitochondrial introgression caused by ongoing hybridization or, alternatively, by maternally inherited symbionts such as *Wolbachia*, which may result in a considerable underestimation of species diversity using DNA barcoding [40]. Although *Wolbachia* infections are common in filaroid nematodes, they seem absent in secernentean nematodes [41] and we are unaware of the infection rate of *Wolbachia* in marine nematodes. The use of *Wolbachia* specific primers in combination with a positive control (containing DNA from infected filaroid nematodes) could reveal whether *Wolbachia* is indeed present in the *Paracanthonchus/Praeacanthonchus* specimens. Interestingly, the nine COI sequences showed a large indel of nine amino acids compared to all other COI sequences. This suggests that we may have amplified a nonfunctional numt. Although numts are often characterized by the occurrence of stop codons, frame shift mutations and indels [42], they may also resemble quite well the original mitochondrial gene from which they may then be difficult to distinguish [23]. Although the number of numt sequences in the nematode genome seems to be rather small [43], [44], the lower rate of evolution of nuclear genes compared to mitochondrial genes may also explain the identical COI sequence in the case of the *Paracanthonchus/Praeacanthonchus*. Clearly, further investigation is required to elucidate the cause of the identical sequences of *Paracanthonchus/Praeacanthonchus*.

Regardless the possible occurrence of mitochondrial introgression or the presence of numts, a strict quality control of the obtained sequences is required for nematodes. Eukaryotic and prokaryotic organisms attached to the cuticula or present in the gut of nematodes will be co-extracted [45]. Diatoms and other microalgae are an important food source for many estuarine nematode species [46]. In view of the limited number of COI sequences available for marine nematodes, a strict quality control of the obtained sequences is essential to build a reliable reference database. Sequences with low signal and/or double peaks in the chromatogram should be removed from the dataset, and abnormal phylogenetic positions or long branches should alert the investigator for possible non-homology with the genuine COI gene. In the case of the Folmer partition, long branches and basal positions in the tree may also be caused by insufficient taxon sampling, but as long as we have no additional sequences from closely related species of *Ascolaimus* and *Araeolaimus*, we were cautious and removed the two sequences from our dataset.

The applicability of COI to recognize [47] and identify closely related parasitic nematode species [37], [38] also holds for marine nematodes. In the present study, 99.5% of all intraspecific comparisons showed genetic distances <0.05, while 99.3% of all interspecific comparisons where >0.05, suggesting that a threshold of 0.05 K2P distance would discriminate most marine nematode species using the I3-M11 partition. Yet, the presence of a barcoding gap strongly depends on the metrics used [48] and on the number of congeneric taxa sampled [49]. For the present study, congeneric comparisons were limited to three genera and involved very closely related cryptic species which may have the smallest interspecific distance possible. On the other hand, this threshold level corresponds remarkably well with that observed for filaroid nematodes (0.048) [38]. The high concordance between taxonomy and COI sequence data suggests that this threshold value will identify closely related and cryptic species in a wide range of nematode species. This is important, since barcoding marine nematodes traditionally uses the 18S or the 28S genes [10], [36] which provide good resolution at the genus and higher taxon level, but low resolution at the species level [5], [14]. Barcoding marine nematodes would clearly benefit from a multilocus approach where the large database of 18S and 28S genes would provide a solid taxonomic framework and where the I3-M11 partition would allow identification to species level.

### Conclusion

A proper molecular toolbox for identifying nematode species should consider as many useful loci as possible, especially when the currently available nuclear loci (18S and 28S) have low resolution at the species level. The amplification across a wide taxonomic range, the ease of sequence alignment and the variability pattern render the I3-M11 partition of COI a good candidate to increase the identification of marine nematode species, provided there is a good reference database. Our results strongly indicate that nematode DNA barcodes should be thoroughly screened to infer their origin and homology state. Furthermore, digital vouchering of nematode specimens prior to molecular analyses is required especially in those studies that are intended to produce barcodes for new nematode species. Only in this way can a reliable reference database be built.

## Supporting Information

Table S1 Overview of marine nematode taxa used for barcoding with COI. (Number) corresponds to the numbers mentioned in figures 1 and 2, (n) number of specimens collected for each species, (locations) are Breskens (B), Paulina (P), Zeedorp (Z), Kruispolderhaven (K), Nieuwpoort (N), or permanent lab cultures (C). Locations between brackets indicate where other specimens of the species have been found. (Sequence length) indicates length of the sequences, lengths in bold were amplified with JB2-JB5GED, a dash indicates ambiguous sequences, blank spaces indicates a lack of PCR product to sequence, italic lengths indicate sequences from previous studies. (Accession numbers) provides the accession numbers for I3-M11 and M1-M6, with numbers in bold taken from previous studies. (Indels) presence of indels in the sequence alignment is indicated by x.
(0.04 MB XLS)

## References

[1] Heip C, Vincx M, Vranken G (1985). The ecology of marine nematodes.. Oceanography and Marine Biology.

[2] Leduc D (2009). Description of *Oncholaimus moanae* sp nov (Nematoda: Oncholaimidae), with notes on feeding ecology based on isotopic and fatty acid composition.. Journal of the Marine Biological Association of the United Kingdom.

[3] De Mesel I, Derycke S, Moens T, Van der Gucht K, Vincx M (2004). Top-down impact of bacterivorous nematodes on the bacterial community structure: a microcosm study.. Environmental Microbiology.

[4] Hamels I, Moens T, Mutylaert K, Vyverman W (2001). Trophic interactions between ciliates and nematodes from an intertidal flat.. Aquatic Microbial Ecology.

[5] De Ley P, De Ley IT, Morris K, Abebe E, Mundo-Ocampo M (2005). An integrated approach to fast and informative morphological vouchering of nematodes for applications in molecular barcoding.. Philosophical Transactions of the Royal Society B-Biological Sciences.

[6] De Mesel I, Derycke S, Swings J, Vincx M, Moens T (2003). Influence of bacterivorous nematodes on the decomposition of cordgrass.. Journal of Experimental Marine Biology and Ecology.

[7] De Mesel I, Derycke S, Swings J, Vincx M, Moens T (2006). Role of nematodes in decomposition processes: Does within-trophic group diversity matter?. Marine Ecology-Progress Series.

[8] dos Santos GAP, Derycke S, Genevois VGF, Coelho L, Correia MTS (2009). Interactions among bacterial-feeding nematode species at different levels of food availability.. Marine Biology.

[9] Postma-Blaauw MB, de Vries FT, de Goede RGM, Bloem J, Faber JH (2005). Within-trophic group interactions of bacterivorous nematode species and their effects on the bacterial community and nitrogen mineralization.. Oecologia.

[10] Bhadury P, Austen MC, Bilton DT, Lambshead PJD, Rogers AD (2006). Development and evaluation of a DNA-barcoding approach for the rapid identification of nematodes.. Marine Ecology-Progress Series.

[11] Floyd R, Abebe E, Papert A, Blaxter M (2002). Molecular barcodes for soil nematode identification.. Molecular Ecology.

[12] Porazinska DL, Giblin-Davis RM, Faller L, Farmerie W, Kanzaki N (2009). Evaluating high-throughput sequencing as a method for metagenomic analysis of nematode diversity.. Molecular Ecology Resources.

[13] Fitch DHA, Bugajgaweda B, Emmons SW (1995). 18S ribosomal-RNA gene phylogeny for some Rhabditidae related to *Caenorhabditis*.. Molecular Biology and Evolution.

[14] Meldal BHM, Debenham NJ, De Ley P, De Ley IT, Vanfleteren JR (2007). An improved molecular phylogeny of the Nematoda with special emphasis on marine taxa.. Molecular Phylogenetics and Evolution.

[15] Derycke S, De Ley P, De Ley IT, Holovachov O, Rigaux A (2010). Linking DNA sequences to morphology: cryptic diversity and population genetic structure in the marine nematode *Thoracostoma trachygaster* (Nematoda, Leptosomatidae).. Zoologica Scripta.

[16] Avise J (1994). Molecular markers, Natural History and Evolution.

[17] Hebert PDN, Ratnasingham S, deWaard JR (2003). Barcoding animal life: cytochrome c oxidase subunit 1 divergences among closely related species.. Proceedings of the Royal Society of London Series B-Biological Sciences.

[18] Hollingsworth PM, Forrest LL, Spouge JL, Hajibabaei M, Ratnasingham S (2009). A DNA barcode for land plants.. Proc Natl Acad Sci U S A.

[19] Huang DW, Meier R, Todd PA, Chou LM (2008). Slow mitochondrial COI sequence evolution at the base of the metazoan tree and its implications for DNA barcoding.. Journal of Molecular Evolution.

[20] Seifert KA, Samson RA, Dewaard JR, Houbraken J, Levesque CA (2007). Prospects for fungus identification using CO1 DNA barcodes, with *Penicillium* as a test case.. Proc Natl Acad Sci U S A.

[21] Erpenbeck D, Hooper JNA, Worheide G (2006). CO1 phylogenies in diploblasts and the ‘Barcoding of Life’ - are we sequencing a suboptimal partition?. Mol Ecol Notes.

[22] Seifert KA (2009). Progress towards DNA barcoding of fungi.. Molecular Ecology Resources.

[23] Song H, Buhay JE, Whiting MF, Crandall KA (2008). Many species in one: DNA barcoding overestimates the number of species when nuclear mitochondrial pseudogenes are coamplified.. Proc Natl Acad Sci U S A.

[24] Folmer O, Black M, Hoeh W, Lutz R, R V (1994). DNA primers for amplification of mitochondrial cytochrome c oxidase subunit I from diverse metazoan invertebrates.. Molecular Marine Biology and Biotechnology.

[25] Derycke S, Backeljau T, Vlaeminck C, Vierstraete A, Vanfleteren J (2007). Spatiotemporal analysis of population genetic structure in *Geomonhystera disjuncta* (Nematoda, Monhysteridae) reveals high levels of molecular diversity.. Marine Biology.

[26] Derycke S, Remerie T, Backeljau T, Vierstraete A, Vanfleteren J (2008). Phylogeography of the *Rhabditis (Pellioditis) marina* species complex: evidence for long-distance dispersal, and for range expansions and restricted gene flow in the northeast Atlantic.. Molecular Ecology.

[27] Bowles J, Blair D, McManus DP (1992). Genetic variants within the genus *Echinococcus* identified by mitochondrial DNA sequencing.. Molecular and Biochemical Parasitology.

[28] Hu M, Chilton NB, Zhu XQ, Gasser RB (2002). Single-strand conformation polymorphism-based analysis of mitochondrial cytochrome c oxidase subunit 1 reveals significant substructuring in hookworm populations.. Electrophoresis.

[29] Derycke S, Remerie T, Vierstraete A, Backeljau T, Vanfleteren J (2005). Mitochondrial DNA variation and cryptic speciation within the free-living marine nematode *Pellioditis marina*.. Marine Ecology-Progress Series.

[30] Galtier N, Gouy M, Gautier C (1996). SEAVIEW and PHYLO_WIN: Two graphic tools for sequence alignment and molecular phylogeny.. Computer Applications in the Biosciences.

[31] Edgar RC (2004). MUSCLE: multiple sequence alignment with high accuracy and high throughput.. Nucleic Acids Research.

[32] Jacob JEM, Vanholme B, Van Leeuwen T, Gheysen G (2009). A unique genetic code change in the mitochondrial genome of the parasitic nematode *Radopholus similis*.. BMC Reserach Notes.

[33] Tamura K, Dudley J, Nei M, Kumar S (2007). MEGA4: Molecular evolutionary genetics analysis (MEGA) software version 4.0.. Molecular Biology and Evolution.

[34] Hebert PDN, Cywinska A, Ball SL, DeWaard JR (2003). Biological identifications through DNA barcodes.. Proceedings of the Royal Society of London Series B-Biological Sciences.

[35] dos Santos GAP, Derycke S, Fonseca-Genevois VG, Coelho L, Correia MTS (2008). Differential effects of food availability on population growth and fitness of three species of estuarine, bacterial-feeding nematodes.. Journal of Experimental Marine Biology and Ecology.

[36] Creer S, Fonseca VG, Porazinska DL, Giblin-Davis RM, Sung W (2010). Ultrasequencing of the meiofaunal biosphere: practice, pitfalls and promises.. Molecular Ecology.

[37] Elsasser SC, Floyd R, Hebert PDN, Schulte-Hostedde AI (2009). Species identification of North American guinea worms (Nematoda: *Dracunculus*) with DNA barcoding.. Molecular Ecology Resources.

[38] Ferri E, Barbuto M, Bain O, Galimberti A, Uni S (2009). Integrated taxonomy: traditional approach and DNA barcoding for the identification of filarioid worms and related parasites (Nematoda).. Frontiers in Zoology.

[39] Casiraghi M, Anderson TJC, Bandi C, Bazzocchi C, Genchi C (2001). A phylogenetic analysis of filarial nematodes: comparison with the phylogeny of *Wolbachia* endosymbionts.. Parasitology.

[40] Whitworth TL, Dawson RD, Magalon H, Baudry E (2007). DNA barcoding cannot reliably identify species of the blowfly genus *Protocalliphora* (Diptera: Calliphoridae).. Proceedings of the Royal Society B-Biological Sciences.

[41] Bordenstein SR, Fitch DHA, Werren JH (2003). Absence of *Wolbachia* in nonfilariid nematodes.. Journal of Nematology.

[42] Zhang DX, Hewitt GM (1996). Nuclear integrations: Challenges for mitochondrial DNA markers.. Trends in Ecology & Evolution.

[43] Bensasson D, Zhang DX, Hartl DL, Hewitt GM (2001). Mitochondrial pseudogenes: evolution's misplaced witnesses.. Trends in Ecology & Evolution.

[44] Richly E, Leister D (2004). NUMTs in sequenced eukaryotic genomes.. Molecular Biology and Evolution.

[45] Bhadury P, Bridge PD, Austen MC, Bilton DT, Smerdon GR (2009). Detection of fungal 18S rRNA sequences in conjunction with marine nematode 18S rRNA amplicons.. Aquatic Biology.

[46] Moens T, Vincx M (1997). Observations on the feeding ecology of estuarine nematodes.. Journal of the Marine Biological Association of the United Kingdom.

[47] Blouin MS (2002). Molecular prospecting for cryptic species of nematodes: mitochondrial DNA versus internal transcribed spacer.. International Journal for Parasitology.

[48] Meier R, Zhang GY, Ali F (2008). The use of mean instead of smallest interspecific distances exaggerates the size of the “barcoding gap” and leads to misidentification.. Systematic Biology.

[49] Jansen G, Savolainen R, Vepsalainen K (2009). DNA barcoding as a heuristic tool for classifying undescribed Nearctic Myrmica ants (Hymenoptera: Formicidae).. Zoologica Scripta.

